# Effectiveness of a school-based, lay counselor-delivered cognitive behavioral therapy for Chinese children with posttraumatic stress symptoms: a randomized controlled trial

**DOI:** 10.1016/j.lanwpc.2023.100699

**Published:** 2023-02-02

**Authors:** Jina Li, Jia Li, Weijun Zhang, Gengchao Wang, Zhiyong Qu

**Affiliations:** a State Key Laboratory of Cognitive Neuroscience and Learning, Beijing Normal University, Beijing, 100875, China; b Center for Behavioral Health & School of Social Development and Public Policy, Beijing Normal University, Beijing, 100875, China; c State Key Laboratory of Liver Research, University of Hong Kong, Hong Kong, 999077, China

**Keywords:** Cognitive behavioral therapy, Lay counselors, Posttraumatic stress disorder, Trauma-affected children, School-based intervention, LMICs, low- and middle-income countries, PCPI, the power up children's psychological immunity, PTSD, posttraumatic stress disorder, PTSD-RI-5, UCLA PTSD Reaction Index for DSM-5, TAU, treatment as usual, TF-CBT, trauma-focused cognitive behavioral therapy, the PCL-5, the PTSD Checklist-5

## Abstract

**Background:**

Improving children's access to mental health services need more innovative solutions, especially in low- and middle-income countries. School-based psychosocial interventions delivered by lay counselors may be an efficient way to improve children's access to mental health services. But few studies were conducted to examine the effectiveness of these interventions. Therefore, this study is to evaluate the effectiveness of trauma-focused cognitive behavioral therapy (TF-CBT) in a group format delivered by lay counselors to children with trauma-related symptoms in China.

**Methods:**

A total of 234 children (aged 9–12 years) with full or subthreshold posttraumatic stress disorder (PTSD) were randomly assigned to group-based TF-CBT or treatment as usual (TAU). In the intervention group, 118 children received 10–12 sessions of group-based TF-CBT delivered by lay counselors for 9 consecutive weeks. In the TAU group, 116 children received the usual school services provided by psychology teachers. The primary outcome was the reduction in PTSD severity, which was assessed with the UCLA PTSD reaction index for DSM-5 (PTSD-RI-5). The secondary outcomes included the reduction in PTSD severity and the remission of PTSD, both of which were measured with the PTSD checklist-5 (PCL-5). Secondary outcomes also included the reduction in depression severity and the reduction in generalized anxiety severity. Blinded assessments were collected at baseline, posttreatment (primary endpoint), and 3-month follow-up. This trial is registered with Chinese Clinical Trial Registry, ChiCTR1900027131.

**Findings:**

At posttreatment, the intervention group scored significantly lower than the TAU group on PTSD-RI-5 PTSD (30.98 vs. 39.22; adjusted mean difference [AMD], −7.35; 95% CI, −11.66 to −3.04), PCL-5 PTSD (28.78 vs. 38.04; AMD, −8.49; 95% CI, −13.23 to −3.75), depression (5.52 vs. 7.96; AMD, −1.63; 95% CI, −2.50 to −0.76), and generalized anxiety (7.23 vs. 8.64; AMD, −1.21; 95% CI, −2.20 to −0.23). The remission of PCL-5 PTSD was also significantly higher in the intervention group (42.86% vs. 13.54%, *χ*^*2*^ = 13.10, *P* < 0.001). These two groups showed a similar level of symptoms at the 3-month follow-up.

**Interpretation:**

The group-based TF-CBT can significantly alleviate PTSD, depression, and generalized anxiety right after treatment in Chinese children who suffer from different types of trauma. But the long-term effects of this intervention need to be further tested. This intervention can be delivered by trained lay counselors in low- and middle-income countries.

**Funding:**

None.

**Research in context**

**Evidence before this study**

Ifigeneia Mavranezouli and colleagues reviewed 32 randomized clinical trials done before January 2018, which examined the relative effectiveness of psychological, psychosocial and other nonpharmacological treatments for PTSD in children and young people. Yajie Xiang and colleagues reviewed 56 randomized clinical trials done before December 2020, which compared the different types and formats of psychotherapies for PTSD in children and adolescents. Both reviews showed that the group-based trauma-focused cognitive behavioral therapy (TF-CBT) was effective in reducing PTSD symptoms among children with PTSD. But no psychosocial intervention has been developed for traumatized children in China. The group-based TF-CBT for traumatized children is also not used often in most low- and middle-income countries (LMICs).

**Added value of this study**

To our knowledge, this is the first large, randomized controlled trial study in China, which uses task-shifting strategies to evaluate the effectiveness of a cultural adaptation of the group-based TF-CBT in traumatized children in school settings. Our study showed that the group-based TF-CBT was more effective than usual care in reducing posttraumatic stress, depression and generalized anxiety in traumatized children. The group-based TF-CBT can be used to treat a group of children who suffer from different types of trauma together. Our study also showed that trained lay counselors, specifically, college students supervised by experts over distance, were able to successfully deliver the group-based TF-CBT to children in school settings.

**Implications of all the available evidence**

The psychological need of traumatized children is huge in China. The group-based TF-CBT intervention is an effective treatment that can be delivered to children who suffer from different types of trauma in China and in other low- and middle-income countries. Building on the evidence that lay counselors can be trained to deliver the group-based TF-CBT intervention, this intervention probably has the potential for scale-up in school settings to reduce the burden from common mental health disorders, especially in LMICs.

## Introduction

Improving children's access to mental health services needs more innovative solutions.^1^ China and other low- and middle-income countries (LMICs) may need more of these solutions. In these countries, traumatic life events are highly prevalent, and these events produce various psychiatric disorders, including posttraumatic stress disorder (PTSD), depression, and anxiety.^2^^,^^3^ But the majority of trauma-affected children are often unable to access mental health care, and children with psychological trauma are most often left untreated.^4^^,^^5^ Untreated psychological trauma can affect children's development and lower their well-being.^6^^,^^7^ In this context, all countries struggle to satisfy these children's mental health needs. There is an increasing demand for treatments that are feasible to be widely implemented in LMICs.

These negative impacts of trauma especially influence Chinese children. In 2020, the child population in China (0–14 years old) reached approximately 253 million, accounting for more than one-tenth of the world's child population.^8^ Nearly half of all children experience traumatic events, and 6.68%–12.65% of these children meet the provisional PTSD diagnosis criteria.^3^^,^^9^^,^^10^ However, only a very small proportion (approximately 9.5%) of people with mental disorders receive any type of mental health treatment in China.^11^ The negative impact of children's mental disorders is further compounded by resource constraints. Therefore, it calls for interventions that address the needs of trauma-affected children in China.

As an effective and cost-effective way to reach larger numbers of children, several group-based cognitive behavioral interventions (group-based CBT) have been developed for trauma-affected children to improve access to mental health services.^12^, ^13^, ^14^, ^15^ There was evidence that supported the effectiveness of group-based CBT for children with psychological trauma.^16^^,^^17^ Until now, some randomized controlled trials have been conducted to examine the effectiveness of these interventions in LMICs. Findings from these studies showed that group-based CBT was more effective than treatment as usual in reducing PTSD symptoms.^18^, ^19^, ^20^ Similarly, some studies suggested that the effect of group-based CBT on PTSD was maintained at the 3-month follow-up and the 12-month follow-up.^15^^,^^20^ But most of these studies were conducted in war-affected or disaster-affected areas, not under normal circumstances.^18^^,^^19^^,^^21^, ^22^, ^23^, ^24^ In these studies, researchers selected a group of children who were exposed to the same type of trauma, such as disaster, parental death, or political violence.^18^, ^19^, ^20^ But children in schools often suffered from different types of trauma. Most importantly, these studies did not examine whether lay counselors were capable of delivering the group-based intervention to a group of children facing different types of trauma. It remains an empirical question whether these group-based psychosocial interventions can be widely used in school settings. Randomized controlled trials of group-based psychosocial interventions to assist trauma-affected children are needed.

To address these critical gaps, the Power up Children's Psychological Immunity (PCPI) program is implemented in China. In this program, trauma-focused cognitive behavioral therapy (TF-CBT) in a group-based format is developed to address children's PTSD in school settings. This intervention is called the PCPI treatment to reduce children's prejudice toward those children who receive the treatment. The development of this intervention is based on the principles of cognitive behavioral theory. It addresses that changes in feelings, cognition, and behavior are critical factors of children's PTSD.^25^ Using a variety of techniques, such as relaxation techniques, trauma narrative, and cognitive processing, can reduce the severity of PTSD in children.^26^ In work preceding this trial, a previous study was conducted to understand the mental health problems of trauma-affected children in China.^27^ This study found that 20.72% of Chinese children who experienced trauma met the criteria for probable PTSD. A previous pilot study was also conducted to examine the cultural appropriateness and feasibility of the PCPI intervention.^28^ The PCPI intervention was found to be feasible as a high retention rate (100%) and an attendance rate of each session (93.33%). Children usually accepted the treatment (i.e., session duration, contents and homework), and their satisfaction with it was high. The PCPI intervention reduces symptoms of PTSD and anxiety right after treatment. However, it is unknown whether this intervention is effective and whether lay counselors are capable of delivering this intervention.

The present trial is designed to assess the effectiveness of the PCPI intervention, specifically examining whether the PCPI intervention delivered by lay counselors was more effective than the treatment as usual (TAU) intervention delivered by psychology teachers in reducing the severity of trauma-related mental health symptoms. We hypothesized that the PCPI intervention would reduce the severity of PTSD and the associated depression and anxiety more than the TAU intervention. We also hypothesized that the rate of PTSD remission in the PCPI group would be higher than the rate of PTSD remission in the TAU group.

## Methods

### Study design

This study was a parallel, two-group, individually randomized, controlled superiority trial, with trial personnel (outcome assessors, data analysts) masked to the participants’ condition assignments. This study was approved by the Chinese Ethics Committee of Registering Clinical Trials (ChiECRCT-20180191) and was registered under the Chinese Clinical Trial Registry (ChiCTR1900027131). Written informed consent was obtained from all of the participants, parents, or guardians of minors. More details about this study protocol can be found in the Appendix (pp 2–11).

### Participants

The participants were school-aged children recruited from six primary schools (four public schools and two private schools) in two cities of Henan Province, China. Recruitment occurred from November 2, 2019, to November 20, 2019. Inclusion criteria were 9–12 years old, history of at least one traumatic event (>one month), and full or subthreshold PTSD (up to one symptom missing) diagnostic criteria from the PTSD Checklist-5 (the PCL-5). Exclusion criteria included: 1) severe psychopathology requiring urgent medical attention; 2) moderate to high suicide risk (e.g., intent or plan to attempt suicide in the near future); 3) showed receptive or expressive language difficulties (written or spoken); 4) currently taking psychotropic medication, such as selective serotonin reuptake inhibitors (SSRI) and serotonin-norepinephrine reuptake inhibitors (SNRI).

Based on our pilot randomized controlled trial, the difference in PTSD severity between PCPI and TAU groups was reported with the effect size (Cohen's *d*) of 0.38.^28^ With regard to the primary outcome after treatment, 110 participants per group were needed to achieve 80% power at a two-sided 5% significance level. Given an anticipated dropout rate of 30%, our target was to recruit a total size of 286 in this study (calculated using G∗Power 3.1).^29^

### Procedures

With the support and assistance of the schools, we conducted a combination of whole-school and classroom-level publicity activities. We introduced the purpose of the study and the content of the survey to children and their caretakers. All children were told that their answers would be kept strictly confidential and would not be disclosed to anyone within or outside of the school. After obtaining informed consent from the caretakers and the children, screening was conducted with school classes as the unit. In this program, we recruited the participants from grades 3 to 5 by selecting children with PTSD symptoms. A self-report questionnaire, including a range of potentially traumatic events and symptoms of PTSD (the PTSD Checklist-5), was handed out and evaluated by trained assessors. For children who met inclusion criteria, they received a brief, in-person interview to assess whether they met exclusion criteria. Afterward, they received a further baseline assessment. They also filled out the demographic information. All participants and their caregivers signed a consent form. For children who were suffering from PTSD symptoms and did not participate in our program, we provided them with some information that might be helpful for them to seek psychological help.

Randomization was performed using computerized software on a 1:1 basis. A random list was generated by using a computer-generated random sequence of numbers in a randomly sized block (2, 4, and 6), stratified by PTSD severity (full PTSD or subthreshold PTSD), and recruitment school sites. An independent research assistant, who was not involved in any other aspect of the study, was responsible for generating this list and placed it in sequentially numbered, opaque, sealed envelopes. After all participants in each school completed assent or consent procedures and baseline assessments, two independent support staffs opened sealed envelopes and recorded the group list. A professor (Z.Y.Q) maintained a master list of identification numbers to enable fidelity checks.

The participants who were randomly assigned to the intervention group received nearly two months of PCPI treatment delivered by lay counselors starting in mid-November 2019. During this period, the TAU children continued to receive their usual school services. Also, children in the intervention group were allowed to receive their usual school services. Both groups of children completed assessments three times: before random assignments (baseline), within one week after treatment (posttreatment), and three months after treatment (3-month follow-up). All outcome assessments were performed in a confidential location via interview format by independent assessors. These assessors, who were blind to participants’ condition assignments, received a 2-day training by an expert in the field of child and adolescent mental health.

### Intervention

The PCPI intervention was developed based on core elements of evidence-based treatment for trauma-affected children—the individual format of TF-CBT.^30^ The specific contents of each session were modified or designed by researchers from the Center for Behavioral Health, Beijing Normal University. More details of the cultural adaptation process can be seen in an article.^28^ Briefly, modifications were made based on Chinese children's characteristics and the implementation contexts, such as reducing the involvement of children's parents, promoting children's intentions and abilities to seek support, modifying the contents of cognitive coping, and changing the treatment entry from communities to school settings.

The PCPI intervention included 7 group sessions and 3 to 5 individual sessions for 9 consecutive weeks. Each group session was held by 2 trained lay counselors at the times that do not have conflicts with children’s studying time. Each group session lasted for about 50 minutes. There were six to eight children in each group. In Session 1, group information, knowledge about trauma, and usual reactions to trauma were explained. In Session 2, children were taught a strategy to deal with stress, specifically, slow breathing and muscle relaxation. In Session 3, emotion regulation is discussed, and regulation strategies were taught. In sessions 4–5, children were taught how to differentiate thoughts, feelings, and behaviors. They were also taught how to stop or change inaccurate or useless thoughts. In Session 6, children were taught how to seek social support when they were under stress. In Session 7, counselors and children reviewed what they had learned in the previous sessions. They also held a closing ceremony. Generally, the individual trauma narrative sessions were conducted from Week 3 to Week 8. Each individual session lasted for approximately 50 minutes. The PCPI manual consisted of 3 sections. It can be completed in 3–5 sessions according to the treatment goals. In individual sessions, the children were encouraged to create a narrative about trauma and to process trauma-related cognitions.

The PCPI intervention was delivered by 15 college students from Henan Normal University. They were recruited from a part-time club in the university through interviews. None of them had previous experience in counseling or delivering psychological treatment. Lay counselors received 3 days of intervention training, 1.5 days of booster training in the midterm intervention, and weekly intensive supervision from three manual developers from the Center for Behavioral Health, Beijing Normal University. The intervention training was conducted in a group. The training contents included explanations on children's trauma experiences and PTSD, the delivery of intervention strategies, and basic counseling skills. After the intervention training, each trainee was asked to take a test of theoretical knowledge and practical skills after the training. 15 lay counselors passed the test and were assigned to six schools based on need. During the intervention phase, they received a detailed instructor manual of the PCPI intervention. In this manual, session-by-session instructions are provided on ways to convey the information that is to be presented to children. They also received a mean of 2.5 h of weekly group supervision via telecommunication software.

With the children's consent, all group sessions were videotaped. The intervention fidelity was monitored by 5 trained observers who watched the video-recorded therapy sessions. The intervention fidelity was measured with a list of items that address the key intervention strategies in each session. The observers checked whether a specific item was covered in a session or not. Individual sessions were not videotaped due to privacy considerations. However, supervisors took steps to make sure of the fidelity of individual sessions. They reviewed the brief reports that were written by lay counselors, discussed the interview details with these counselors, and addressed the barriers that the counselors encountered. If the counselors did not address some contents that they should have addressed, they were asked to discuss these contents in the next session.

### Treatment as usual (TAU)

Children in the TAU group continued to receive mental health services that were delivered by their psychology teachers. These services mainly included mental health courses, organized mental health activities, and individual counseling. Mental health courses covered six topics, including understanding oneself, emotion regulation, learning habits, interpersonal skills, personality development, and adaption to personal life and society. These courses lasted 18 weeks, and they were provided twice a week. Organized mental health activities were held once a month, including providing mental health knowledge to students, solving their psychological crisis before an exam, and preventing their psychological crisis. Additionally, the school set up a counseling room. Individual counseling were provided to children in need if they sought support. Their psychology teachers used multiple catharsis techniques to guide children to reduce the tension of their suppressed emotions. Their intervention was not focused on traumatic events. They only talked with children about trauma when children proposed to discuss it. These psychology teachers participated regularly in the local government training programs, but they were not provided any ongoing supervision. For ethical reasons, these psychology teachers were visited twice a month by researchers in the study to assess the children's conditions. If the children had major psychological problems, they were referred to professional mental health clinics. During the period of study, no child was referred to a clinic or hospital due to psychological problems.

### Outcome measurements

The primary outcome was changes in PTSD severity measured with the UCLA PTSD Reaction Index for DSM-5 (PTSD-RI-5) from baseline to posttreatment. The secondary outcomes were changes in children's PTSD severity and PTSD remission measured with the PTSD Checklist-5 (PCL-5), children's depression severity measured with the Children Depression Inventory-Short (CDI-S), and children's generalized anxiety severity measured with the subscale of Screen for Child Anxiety Related Emotional Disorders (SCARED) from baseline to posttreatment and 3-month follow-up.

### Primary outcome

PTSD-RI-5 was used to screen for trauma exposure and assess DSM-5 PTSD symptoms.^31^ Part one of the scale included 13 potentially traumatic experiences. Part two consisted of 27 items to assess PTSD symptoms and 4 additional items to assess the dissociative subtype. All items were rated from 0 (none) to 4 (almost every day), and higher scores indicated higher levels of PTSD severity.^31^^,^^32^ The cutoff point was 35 for a provisional diagnosis of PTSD.^33^ The PTSD-RI-5 was translated into Chinese with the translation-back translation approach, and each word was checked for conceptual understanding and understandability by Chinese children. The internal consistency was high in our sample (Cronbach's α = 0.91).

### Secondary outcomes

PCL-5 was a 20-item self-report scale that assesses PTSD symptoms. Items were rated on a 5-point Likert scale from 0 (“not at all”) to 4 (“extremely”). There were two ways to diagnose full PTSD: 1) a child had a total score that was greater than or equal to 33. 2) a child showed at least moderate severity for each of the four symptom clusters. This meant that they chose one or more B items (items 1–5), one or more C items (items 6–7), two or more D items (items 8–14) and two or more E items (items 15–20).^34^ If a child did not meet the DSM-5 PTSD criterion but endorsed three of the four symptom clusters, he/she was diagnosed with subthreshold PTSD.^35^^,^^36^ The Chinese version of PCL-5 was adapted by translation and back translation. It has shown good psychometric properties in trauma-affected Chinese children.^27^ In the present study, the PCL-5 was also used to assess the children's PTSD remission because it was the main screening tool that was used to recruit children. Remission was defined as no longer meeting the diagnostic criteria of full PTSD.^34^ The internal consistency was also good in our sample (Cronbach's α = 0.82).

CDI-S was used to assess the severity of children's depressive symptoms.^37^ Higher scores indicated more depressive symptomatology. Each item was scored on a three-point scale, ranging from 0 (“no symptoms”) to 2 (“serious symptoms”). A sum score of 7 was identified as the optimal screening cut-off score.^38^ The 10-item scale shows high internal consistency in our sample (Cronbach's α = 0.84).

The subscale of SCARED was used to assess the children's generalized anxiety disorder.^39^ Children rated the frequency of each symptom on a 3-point scale, ranging from 0 (“not true”) to 2 (“very true”). A sum score of 7 was identified as the optimal screening cut-off score.^40^ The 9-item scale had acceptable internal consistency in our sample (Cronbach's α = 0.78).

### Protocol changes

Three main changes were made to the protocol. First, a TAU group was used instead of a waiting-list control group. Our trial schools commonly provided various forms and amounts of mental health services that could affect the children's mental health. Limiting the children's participation in these services seemed to be unreasonable, so the TAU intervention was selected instead. Second, 3- and 6-month follow-up assessments using the face-to-face format were initially planned. However, one month after the treatment, the COVID-19 pandemic broke out in China. COVID-19 had adverse psychological effects on the children, which affected the effectiveness of the present study to a large extent. Considering the difficulty of visits in person, the data safety and monitoring committee made recommendations, and the investigators decided to stop this trial after the 3-month follow-up assessment. Additionally, our total sample size was changed from 286 to 234 to accommodate the low dropout rate and the impossibility of delivering treatment in person in school settings. In the present study, the dropout rate of 5.13% was lower than expected. The remaining analysis size, 222 children, was larger than the power calculated size without dropout, specifically, 220 children. Third, the age range of participants was changed from 7–15 years old to 9–12 years old. Based on our experience of running the pilot study, children aged 7–8 years may not understand the treatment materials well, so children aged 7–8 years were not recruited in the present study. Additionally, we only recruited children in elementary schools this time, so children aged 13–15 years, who were in secondary schools, were not included.

### Statistical analyses

The data were analyzed with the intention-to-treat approach. For the participants' loss to follow-up and each missing item, the missing data were imputed 5 times by multiple imputation procedures.

To determine whether participants in the PCPI and TAU groups manifested different patterns of change in PTSD-RI-5 PTSD symptoms over time, a linear mixed-effects regression was used. The fixed effects included the treatment condition, the time, and their interaction; the baseline measurement was a covariate; and the subject-level variabilities were treated as random effects. The adjusted mean difference and 95% of it at each time point between the two groups were derived from the mixed model. Between-group effect sizes were calculated for each outcome according to Cohen's *d* statistic. This analytic approach was also used for secondary outcomes, including PCL-5 PTSD scores, depression scores and generalized anxiety scores. Additionally, χ^2^ tests were used to examine the difference in the PCL-5 PTSD remission rate between the PCPI group and TAU group. Given that the participants had a wide range of PTSD-RI-5 PTSD scores at the baseline level, post hoc subgroup analyses were also performed using the interaction terms between the randomized arm and the baseline primary severity (<35 points vs. ≥35 points).

Sensitivity analyses were conducted to examine the robustness of the effect of the present findings. First, only the data of children who completed all of the scales and who did not have any missing values were included in the analysis to avoid possible inconsistencies due to the use of the imputation method. Second, to examine whether the data of children with subthreshold PTSD influenced the main finding, we did a sensitivity analysis using the data of children with full PTSD. All statistical analyses were conducted in R version 3.6.1 by an independent trial statistician who did not know the treatment assignment.

### Role of funding source

The funder of the present study had no role in the study design, data collection, data analysis, data interpretation, or writing of the report. The corresponding author had full access to all data in the study and had responsibility for the decision to submit the manuscript for publication.

## Results

**Fig. 1:**
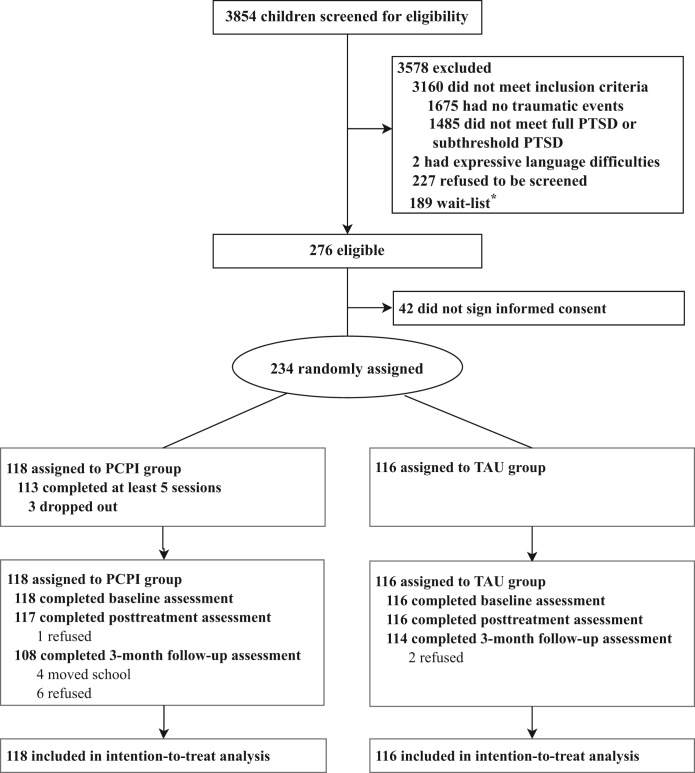
**Trial Profile.** PTSD indicates posttraumatic stress disorder; PCPI, the power up children's psychological immunity; TAU, treatment as usual. ∗189 children met subthreshold PTSD, but were not able to be scheduled on the treatments due to a shortage of lay counselors or conflicts with the schedules of their school work.

Of 3854 children screened, 234 children were eligible and agreed to participate. These children were randomly assigned to the PCPI (n = 118) or TAU group (n = 116) (Fig. 1). In the PCPI group, two children dropped out after the first group session, and one child dropped out after the third group session. These three children were invited to complete follow-up assessments. Of the 115 remaining children, 113 participated in five or more group sessions, and 114 participated in 2–5 individual sessions. In terms of the outcome data, 233 children (99.57%) completed the posttreatment assessment, and 222 (94.87%) completed the 3-month follow-up assessment. The children who completed all the assessments did not differ significantly in the baseline variables from those who did not complete all the assessments (Table S1; Appendix pp 12).

In this study, 105 group sessions were delivered by 15 lay counselors. 100 of these sessions were videotaped to assess the treatment fidelity. Five sessions were not videotaped due to equipment problems. Overall, the results showed good content fidelity (Table S2; Appendix pp 13). The mean content fidelity score for each session was above 80%, and 72 out of 100 sessions (72%) were rated as having excellent content fidelity (>85%).

The demographic characteristics and traumatic events were similar across the groups (Table 1). Among the 234 children, the mean (SD) age was 10.41 (0.88) years. A total of 137 (58.55%) were boys, 122 (52.14%) were in the fifth grade, 93 (39.74%) were boarders, and 40 (17.09%) had subthreshold PTSD. Bereavement (55.13%) was the most common type of traumatic experience. Medical treatment (52.99%), animal attacks (47.44%), bad accidents (47.01%), and domestic violence (46.58%) were the next most common.

**Table 1:** Demographic characteristics and traumatic events of children in PCPI and TAU.^a^

Characteristic	PCPI (n = 118)	TAU (n = 116)	Total (N = 234)
**Age, mean (SD)**	10.41 (0.88)	10.42 (0.89)	10.41 (0.88)
**Sex**			
Boy	67 (56.78%)	70 (60.34%)	137 (58.55%)
Girl	51 (43.22%)	46 (39.66%)	97 (41.45%)
**School grade**			
3	9 (7.63%)	10 (8.62%)	19 (8.12%)
4	45 (38.14%)	48 (41.38%)	93 (39.74%)
5	64 (54.24%)	58 (50.00%)	122 (52.14%)
**Parent's marital status**			
Married or remarried	93 (78.81%)	94 (81.03%)	187 (79.91%)
Single or widowed	25 (21.19%)	22 (18.97%)	47 (20.09%)
**Boarders (living in school)**	49 (41.53%)	44 (37.93%)	93 (39.74%)
**Subthreshold PTSD (the PCL-5)**	20 (16.95%)	20 (17.24%)	40 (17.09%)
**Trauma experience**			
A disaster (earthquake, wildfire, and flood, etc.)	22 (18.64%)	19 (16.38%)	41 (17.52%)
A bad accident (a serious car accident or fall)	49 (41.53%)	61 (52.59%)	110 (47.01%)
Hit, punched, or kicked at home	50 (42.37%)	55 (47.41%)	105 (44.87%)
Witnessing domestic violence	51 (43.22%)	58 (50.00%)	109 (46.58%)
Beaten up or threatened in school	47 (39.53%)	52 (44.83%)	99 (42.31%)
Serious injury or violent death of a loved one or friend	36 (30.51%)	48 (41.38%)	84 (35.90%)
A painful or scary medical treatment	57 (48.31%)	67 (57.76%)	124 (52.99%)
Anyone close to you died	66 (55.93%)	63 (54.31%)	129 (55.13%)
Seeing a dead body	47 (39.83%)	49 (42.24%)	96 (41.03%)
Touched private parts or sexual abuse	10 (8.47%)	13 (11.21%)	23 (9.83%)
Someone was beaten up, shot at, or killed	21 (17.80%)	26 (22.41%)	47 (20.09%)
Sudden separation with a loved one	46 (38.98%)	59 (50.86%)	105 (44.87%)
Beaten or attacked by an animal	56 (47.46%)	55 (47.41%)	111 (47.44%)

PCPI, the power up children's psychological immunity; TAU, treatment as usual; PTSD, posttraumatic stress disorder.

a Data are presented as number (percentage) unless otherwise indicated.

### Primary outcomes

**Fig. 2:**
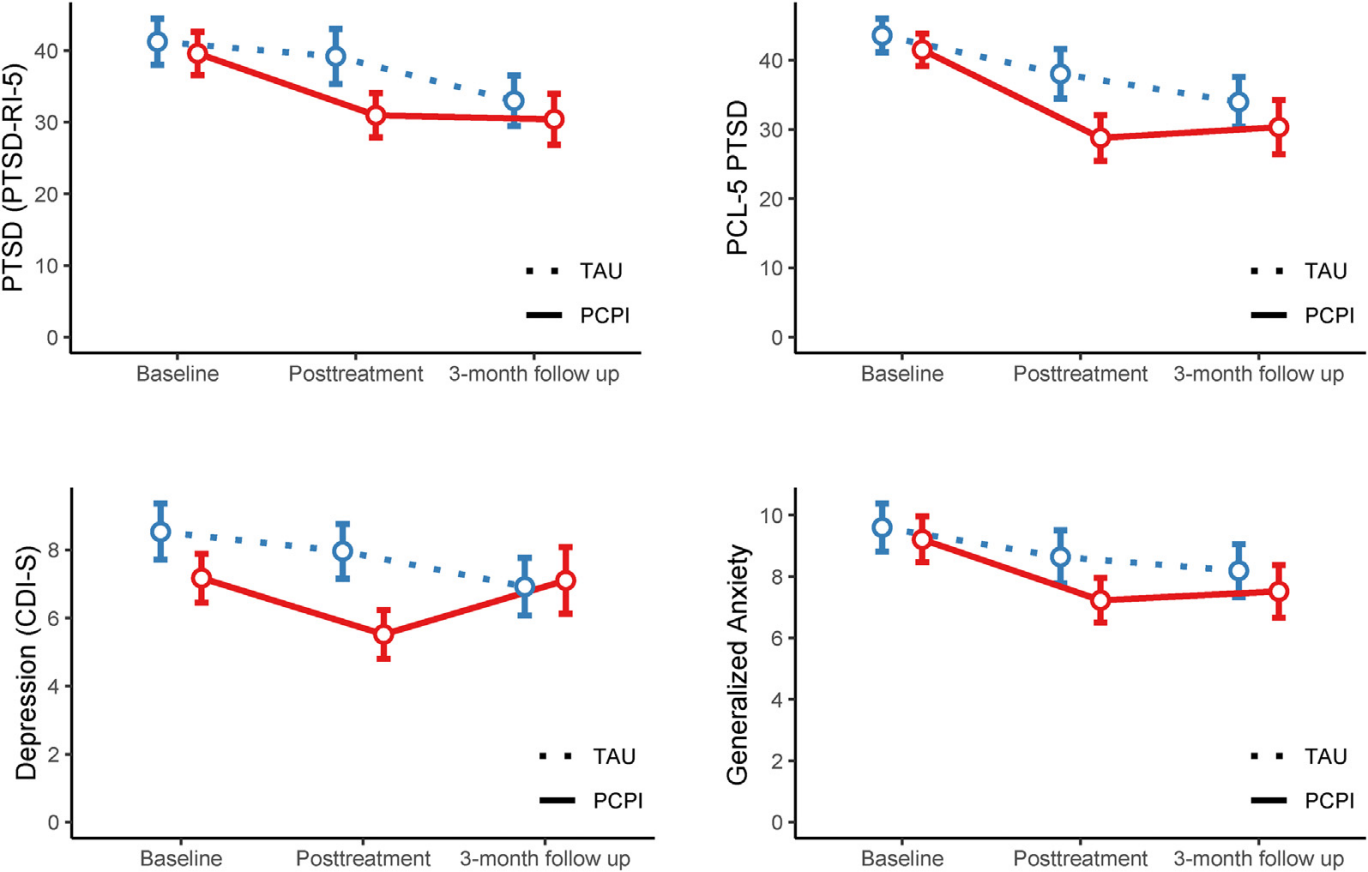
**Treatment effects of the PCPI group and the TAU group.** PTSD indicates posttraumatic stress disorder; PCPI, the power up children's psychological immunity; TAU, treatment as usual; PTSD-RI-5, PTSD Reaction Index for DSM-5; PCL-5, PTSD Checklist-5; At posttreatment, the PCPI group had a significantly lower level of symptoms of PTSD, depression, and generalized anxiety than the TAU group; At the 3-month follow-up, these two groups showed a similar level of symptoms.

Table 2 and Fig. 2 showed the results of the primary and secondary outcomes that were measured at three points in the study. At the baseline, the PCPI group and the TAU group had similar scores on PTSD-RI-5 PTSD (mean [SD], 39.63 [16.66] vs. 41.28 [17.75]; adjusted mean difference [AMD], −1.66; 95% CI, −6.07 to 2.76). At posttreatment, the PCPI group had a significantly lower PTSD-RI-5 PTSD score than the TAU group (mean [SD] score, 30.98 [17.22] vs. 39.22 [21.10]; AMD, −7.35; 95% CI, −11.66 to −3.04). At the 3-month follow-up, the PCPI group had a lower PTSD-RI-5 PTSD score than the TAU group, but the differences were not significant (mean [SD] score, 30.42 [19.83] vs. 33.01 [19.39]; AMD, −2.03; 95% CI, −6.85 to 2.79). Linear mixed analyses were conducted for the primary outcome at three time points (baseline, posttreatment, and 3-month follow-up). The PTSD-RI-5 PTSD scores decreased in both groups over time, with a significantly larger decrease in PTSD-RI-5 PTSD scores for the PCPI group compared with the TAU group (treatment × time interaction, 5.65; 95% CI, 0.90 to 10.40; *P* = 0.02) (Fig. 2).

**Table 2:** Primary and secondary outcomes at three assessment points.

	PCPI (n = 118)	TAU (n = 116)	Adjusted mean difference or odds ratio (95% CI)	*P-*value	Effect size, Cohen's *d*
**Primary outcome**					
PTSD (PTSD-RI-5), mean (95%CI)					
Baseline	39.63 (36.62–42.63)	41.28 (38.05–44.51)			
Posttreatment	30.98 (27.87–34.09)	39.22 (35.38–43.06)	−7.35 (−11.66–−3.04)	0.001	−0.38
3-month follow-up	30.42 (26.84–34.00)	33.01 (29.48–36.54)	−2.03 (−6.85–2.79)	0.41	−0.10
**Secondary Outcome**					
PTSD (PCL-5), mean (95%CI)					
Baseline	41.51 (39.16–43.85)	43.60 (41.16–46.04)			
Posttreatment	28.78 (25.47–32.09)	38.04 (34.47–41.61)	−8.49 (−13.23–−3.75)	<0.001	−0.45
3-month follow-up	30.33 (26.40–34.26)	33.98 (30.40–37.57)	−3.07 (−8.34–2.19)	0.25	−0.15
Depression (CDI-S), mean (95%CI)					
Baseline	7.17 (6.45–7.89)	8.53 (7.71–9.36)			
Posttreatment	5.52 (4.80–6.24)	7.96 (7.15–8.76)	−1.63 (−2.50–−0.76)	<0.001	−0.39
3-month follow-up	7.10 (6.13–8.08)	6.92 (6.07–7.77)	0.73 (−0.50–1.97)	0.25	0.15
Generalized anxiety disorder (the subscale of SCARED), mean (95%CI)					
Baseline	9.21 (8.47–9.96)	9.60 (8.82–10.38)			
Posttreatment	7.23 (6.51–7.95)	8.64 (7.77–9.51)	−1.21 (−2.20–−0.23)	0.02	−0.28
3-month follow-up	7.52 (6.66–8.37)	8.19 (7.33–9.05)	−0.54 (−1.70–0.62)	0.36	−0.11
Meet diagnosis of full PTSD (PCL-5), n (%)					
Baseline	98 (83.05%)	96 (82.76%)	1.02 (0.52–2.02)		–
Posttreatment	56 (47.46%)	83 (71.55%)	0.36 (0.21–0.62)	<0.001	–
3-month follow-up	57 (48.31%)	71 (61.21%)	0.59 (0.35–1.00)	0.06	–

PCPI = the power up children's psychological immunity; TAU = treatment as usual; PTSD-RI-5 = PTSD Reaction Index for DSM-5 (total score range 0–80; higher scores indicated severe posttraumatic stress disorder severity); CDI-S = Children Depression Inventory-Short (total score range 0–20; higher scores indicated severe depression severity); SCARED = The Screen for Child Anxiety Related Emotional Disorders (subscale score range 0–18; higher scores indicated severe generalized anxiety disorder severity); PCL-5 = PTSD Checklist-5 (total score range 0–80; higher scores indicated severe posttraumatic stress disorder severity).

The results of the post hoc subgroup analyses showed that the effect of the intervention was larger for children with higher baseline PTSD-RI-5 PTSD scores (≥35 points) than for those with the lower scores (<35 points) at posttreatment (interaction coefficient, −7.56; 95% CI, −13.86 to −1.27; *P* = 0.02), but not at the 3-month follow-up (interaction coefficient, −2.63; 95% CI, −9.77 to 4.50; *P* = 0.47). The children with higher baseline PTSD-RI-5 scores (≥35 points) profited more from the PCPI intervention at posttreatment. However, no differences were found between the two groups for the children with the lower scores (<35 points) (Table S3; Appendix pp 14).

### Secondary outcomes

The PCL-5 PTSD severity showed the same pattern of improvement as the PTSD-RI-5 PTSD severity at posttreatment (mean [SD] score, 28.78 [18.36] vs. 38.04 [19.62]; AMD, −8.49; 95% CI, −13.23 to −3.75) and the 3-month follow-up (mean [SD] score, 30.33 [21.78] vs. 33.98 [19.69]; AMD, −3.07; 95% CI, −8.34 to 2.19). The PCL-5 PTSD scores decreased in both groups over time, with a significantly larger decrease in PCL-5 PTSD scores for the PCPI group compared with the TAU group (treatment × time interaction, 5.61; 95% CI, 0.59 to 10.63; *P* = 0.03) (Fig. 2).

We also examined the percentage of children who met the PCL-5 full PTSD diagnosis (Table 2). At posttreatment, the number of children who met the PCL-5 full PTSD diagnosis criteria in the PCPI group decreased from 98 to 56 (42.86% remission), whereas the number of children in the TAU group decreased from 96 to 83 (13.54% remission). Children in the PCPI group were more likely to reach PTSD remission than children in the TAU group (OR = 0.36, χ^2^ = 13.10, *P* < 0.001). At the 3-month follow-up, the rate of PTSD remission in the PCPI group was 41.84%, whereas the rate of PTSD remission in the TAU group was 26.04% (OR = 0.59, χ^2^ = 3.43, *P* = 0.06).

The children in the PCPI intervention showed significant improvement in the other secondary outcomes. At posttreatment, mean depression (mean [SD] score, 5.52 [3.99] vs. 7.96 [4.41]; AMD, −1.63; 95% CI, −2.50 to −0.76) and generalized anxiety (mean [SD] score, 7.23 [4.00] vs. 8.64 [4.78]; AMD, −1.21; 95% CI, −2.20 to −0.23) were significantly lower in the PCPI than in the TAU treatment. At the 3-month follow-up, there were no significant mean differences in the depression (mean [SD] score, 7.10 [5.40] vs. 6.92 [4.67]; AMD, 0.73; 95% CI, −0.50 to 1.97) and generalized anxiety scores (mean [SD] score, 7.52 [4.73] vs. 8.19 [4.72]; AMD, −0.54; 95% CI, −1.70 to 0.62) between these two groups. The detailed results can be seen in Table 2 and Fig. 2. The sensitivity analyses did not qualitatively affect our results (Table S4; Appendix pp 15). If only the children with full PTSD were included, the observed decreases in generalized anxiety became nonsignificant.

## Discussion

To our knowledge, this is the first large, randomized controlled trial of a group-based TF-CBT intervention (called the PCPI intervention) conducted in China. It is an intervention delivered by trained lay counselors. It is designed for trauma-affected children in China. Similar to the procedure of other group-based interventions that address trauma, the PCPI intervention guides children to complete the contents of group sessions and discuss trauma narratives in individual sessions.^20^^,^^26^ In sum, the effectiveness of the PCPI intervention was evaluated in school settings. The results of the present study showed that the effect of PCPI intervention that was delivered by trained lay counselors was better than the TAU intervention at posttreatment that was delivered by psychology teachers in reducing the severity of PTSD and the rate of PTSD remission. Even though the severity of their PTSD did not decrease at the 3-month follow-up, it did not return to its previous level. The present finding was consistent with the previous finding that group-based TF-CBT was effective in treating children and young people with PTSD.^15^^,^^16^^,^^22^ It supported the effectiveness of the implementation of group-based TF-CBT in the population of Chinese children.

The present study showed that more children receiving PCPI intervention entered remission than did those receiving TAU at posttreatment (42.86% vs 13.54%, respectively). Furthermore, the remission rate of PTSD in the PCPI group was maintained at the 3-month follow-up. This finding was in line with a recent meta-analysis study investigating remission rates following CBT for anxiety-related disorders in children and adolescents. It found that the overall remission rate was 49.4% in the intervention group and 17.8% in the control group.^41^ This finding was also in line with another meta-analysis study investigating remission rate in CBT for adult anxiety disorders.^42^ This finding is important for a critical evaluation of the success of the PCPI intervention. If the remission rate is short-term, it needs to improve the durability of this intervention.^43^ While this finding is promising, there is significant room for improvement as more than half of children with PTSD remain symptomatic after treatment. It highlights the importance of shifting our focus from statistically significant change to achieving symptom remission.

The present findings also suggested that PCPI intervention may be effective in reducing anxiety and depression in children with PTSD. This finding was consistent with previous studies of the TF-CBT effects on reducing multiple psychosocial issues in children with PTSD.^12^^,^^44^^,^^45^ Given that children with PTSD often have other symptoms,^46^^,^^47^ therapists may have faced challenges preselecting suitable patients when they conduct group-based treatment in routine services. The effectiveness of PCPI on PTSD, anxiety, and depression makes it easier for therapists to provide group-based intervention. So these findings are particularly important because PCPI intervention provides an effective treatment to these children.

The present research may have unique contribution to the field of cognitive behavioral therapy. In most of previous studies on group-based treatments of PTSD, all children suffered from the same type of major trauma.^18^, ^19^, ^20^ However, most of children in the present study experienced complex traumatic events, such as domestic violence, school violence, bereavement, etc. Although complex traumatic events occur more frequently than the traumas related to disaster and sexual harassment among children under normal circumstances in China or other LMICs,^3^^,^^27^^,^^48^ treating children with these traumas may be difficult because these traumas tend to occur repeatedly and chronically. The results of the present study show that children with psychological distress caused by a wide range of traumatic events can benefit from this intervention.

The present finding suggests that lay counselors can provide manualized psychological services to the primary school settings in China and other LMICs. Such positive results can be explained in two ways. First, PCPI includes many known active factors, such as relaxation skills, trauma narratives, cognitive coping skills, and social support skills.^49^, ^50^, ^51^ These active factors not only alleviate PTSD symptoms, but also alleviate other comorbid mental health problems.^52^^,^^53^ Second, the lay counselors were competent and showed fidelity to intervention protocols throughout the study. This ensured the effectiveness of the PCPI intervention. In the present trial, we used a series of strategies to ensure treatment fidelity, including recruiting lay counselors seriously, using a detailed operational manual, training and supervising them regularly. The treatment fidelity in the present trial show that lay counselors can deliver psychological intervention to children effectively under supervision. The present finding provides a promising way of solving understaffing issues in the field of psychological therapy in LMICs.

Further research can be conducted to address the effects of PCPI on PTSD. First, the effect size of our intervention was smaller than the effect size of some TF-CBT in the literature.^54^, ^55^, ^56^ It should be noted that there are two major differences between our study and the previous studies that have large effect sizes. One is that we use group-based intervention, instead of individual intervention. The other is that lay counselors, instead of professionals, delivered the intervention. Furthermore, the lay counselors in the present study were only trained for five days. Besides these reasons, it was also possibly because children in the present study showed a wide range of baseline PTSD scores, ranging from subthreshold to full PTSD. Prior research shows that children's state resists change if they are low in symptom severity.^57^ Our findings were consistent with this explanation. The effect size of our treatment was larger for children with high baseline PTSD-RI-5 PTSD scores. However, it should be noted that these results were post hoc subgroup analyses. Due to these analyses' limitations, future research should be conducted to verify this interpretation of our findings.

Second, children in the PCPI group and those in the TAU group did not differ in their symptoms and outcomes at the 3-month follow-up. This finding is inconsistent with the findings in the literature.^58^^,^^59^ In the present study, only children in the TAU group improved their state from posttreatment to the 3-month follow-up. One possibility was that emotional catharsis techniques used in the TAU group were effective positive ways in alleviating children's distress. But children in the TAU group needed to receive these techniques for a longer time to work.^60^^,^^61^ Another possibility was that the COVID-19 pandemic imposed a serious psychological burden on children.^62^ During this period, children were in similar circumstances, were quarantined at home, and were exposed to a large amount of negative news. As a result, they may suffer from similar psychological problems. Even though the results of the present study were influenced by COVID-19 pandemic, children in the PCPI group recovered faster than those in the TAU group. They reached a level of recovery at posttreatment. But children in the TAU group reached the same level of recovery at 3-month follow-up. It should also be noted that the rate of PTSD remission in the PCPI group was significantly higher than the rate of PTSD remission in the TAU group at posttreatment and the 3-month follow-up. These findings were important because children who are affected by trauma may experience negative developmental consequences if they did not recover.^63^^,^^64^

The present study has important strengths. For example, lay counselors are trained to deliver the intervention. Participants suffer from a wide range of different types of commonly occurring trauma. A few limitations should be mentioned. First, the follow-up period was relatively short, and results from the 3-month follow-up assessment should be interpreted with caution due to the COVID-19 pandemic. Second, the participants in the present study were students in grades 3 to 5. Future research should be conducted to examine whether this intervention can be applied to the treatment of younger or older children. Third, our comparison group was the TAU group instead of an active alternative intervention group because there was no other evidence-based intervention for PTSD in China when the present study was conducted. Fourth, even though assessors were masked at assessment, children may be aware of their own experimental conditions. This may result in expectation biases.

### Conclusions

The present findings suggest that the PCPI intervention is an effective group-based treatment to trauma-affected children in China. It can treat a group of children who suffer from different types of commonly occurring trauma. It can be used to reduce trauma-related symptoms, including PTSD, depression, and generalized anxiety. Furthermore, the PCPI intervention can be delivered by lay counselors. This may provide an effective strategy to promote children's access to mental health services in China. However, further research on the long-term effects of the PCPI intervention is needed.

## Contributors

Jina Li conceived the study, developed the research design and wrote the first draft of the manuscript. Jia Li provided training and supervision for the lay counselors. Weijun Zhang contributed to the design of the trial and commented on the paper. Gengchao Wang did the statistical analyses. Zhiyong Qu planned the study, secured the funding, managed the trial as the principal investigator, and critically revised the manuscript. All authors contributed to and approved the final manuscript.

## Data sharing statement

Anonymised data will be made available upon reasonable request, which must include a protocol and statistical analysis plan and must not be in conflict with our prespecified publication plan. All data requests should be submitted to the corresponding author for consideration. Access to anonymised data, the statistical analysis plan and analytical code will be made available following publication.

## Declaration of interests

All authors declare no competing interests.
