# Supplementary material for: Effectiveness of a school-based, lay counselor-delivered cognitive behavioral therapy for Chinese children with posttraumatic stress symptoms: a randomized controlled trial
Source: Lancet Reg Health West Pac. 2023 Feb 2;33:100699. doi: 10.1016/j.lanwpc.2023.100699 (PMC9918421; doi:10.1016/j.lanwpc.2023.100699)
Supplement: Supplementary Appendix [file mmc1.docx]

**Supplementary Appendix**

[**Study Protocol 2**](#_Toc1211620854)

[**Supplementary Results 12**](#_Toc1271752324)

[Table S1. Baseline characteristics by completion of three timepoint assessments. 12](#_Toc436570877)

[Table S2. The content fidelity for each group session. 13](#_Toc1642591587)

[Table S3. Primary outcomes at three assessment points for different PTSD severity groups. 14](#_Toc1134520524)

[Table S4. Sensitivity analyses for all participants, participants without missing values, and participants with full PTSD. 15](#_Toc379145155)

# Study Protocol

**The Effectiveness of Group-based Trauma-Focused Cognitive Behavioral Therapy (TF-CBT) in Chinese Children: A Randomized Controlled Trial Study Protocol**

**Abstract**

**Introduction:** Although the effectiveness of the group-based trauma-focused cognitive behavioral therapy (the group-based TF-CBT) has been verified in several studies conducted in Western countries, it remains unknown whether it would be effective in Chinese cultural contexts. It is also unknown why and how this treatment works. Therefore, the effectiveness and mechanisms of the group-based TF-CBT should be evaluated among Chinese children exposed to trauma.

**Methods and analysis:** All recruited participants are randomly assigned to the TF-CBT group and the waiting list (WL) group. During the intervention period, service providers receive TF-CBT training and weekly supervision. Primary and secondary outcomes, potential moderators, and mediators will be evaluated using self-reported questionnaires, and assessments are conducted at baseline, 4 weeks during the intervention phase, 2 weeks posttreatment (primary endpoint), 3-month, and 6-month post-end-of-treatment follow-up assessments by independent data assessors. The intent-to-treat (ITT) approach is employed, as needed.

**Ethics and dissemination:** The study has been reviewed and approved by the Ethics Committee of Chinese Clinical Trial Registry. The results will be actively disseminated through peer-reviewed journals, conference presentations, social media, the internet, and community engagement activities.

**Trial registration number:** ChiCTR1900027131

**1. Introduction**

Posttraumatic stress disorder (PTSD) is a common chronic disorder after a traumatic event. It substantially impairs an individual’s quality of life, social functioning, and academic levels.^1^ It is reported that 39·7% have experienced traumatic events, and the prevalence of probable PTSD is 12·65% in school-aged children in China. ^2^ A higher prevalence of PTSD is also found in Palestine, about 34·1%. ^3^ If left untreated, PTSD symptoms may be kept unchanged or become worse over time. So PTSD has been a serious public health challenge. Unfortunately, many people with common mental disorders do not receive treatment, or they receive ineffective treatment in low- and middle-income countries (LMICs). ^4,5^ Especially in some East Asian countries, such as China, Japan, Korea, and Vietnam, families and schools always pay more attention to children’s academic performance and ignore their psychological needs. So parents and teachers often fail to recognize the presence of possible mental health problems in children. As a result, children may not get timely treatment. Compared with other mental disorders, PTSD receives much less attention. Children with PTSD are often left untreated. Therefore, early detection and effective treatment for children’s PTSD are urgent needs in East Asian countries.

Several treatments are used to treat PTSD, including Trauma-focused cognitive behavioral therapy (TF-CBT), Cognitive Processing Therapy (CPT), Prolonged Exposure (PE), and Narrative Exposure Therapy (NET).^6-9^ Among these treatments, TF-CBT has been proven to be the most effective and applied most often in alleviating PTSD among children and adolescents.^10^ A study showed that core components (exposure and cognitive-restructuring techniques) of TF-CBT were used to reduce the distress associated with the traumatic experience, and a series of skills are designed to help children develop coping ability.^9^ These components are useful for children regardless of whether they experienced trauma or not. Recently, TF-CBT has been developed as a group treatment.^11^ Due to the increased demand for mental health services and promoted concern for cost-effectiveness, the group format may be more suitable for LMICs. However, since the group-based TF-CBT protocols of these trials are mainly designed for children in Western cultural contexts, it remains unknown whether it would be effective for those in other cultural contexts, such as East Asian culture. As we all know, contextual factors and barriers, such as variations in explanatory models, and ways of coping with distress, may impede children’s engagement with mental health services, resulting in a negative influence on treatment outcome.^12^ Hence, cross-cultural validation is essential for the application of the group-based TF-CBT in East Asian countries.

So far, several studies have verified the effectiveness of the group-based TF-CBT intervention among children who are exposed to traumatic events.^13,14^ However, the evidence that sheds light on the group-based TF-CBT’s mechanism is insufficient, and little is known about why and how the group-based TF-CBT intervention works. As we all know, not all individuals respond favorably to this treatment. Results about trajectories have shown that 21% of children have no significant improvement in PTSD symptoms over time after they receive TF-CBT.^15^ This suggested that we should understand better the actual processes through which the group-based TF-CBT intervention works. Furthermore, because the group-based TF-CBT protocol in this study has been culturally modified, examining treatment mechanisms can further provide evidence for its effectiveness in Chinese children. Understanding the mechanisms of the treatment will also help us refine existing protocols and reshape the treatment.

In sum, the primary aim of this RCT study is to test the effectiveness of the group-based TF-CBT intervention. We hypothesize that the group-based TF-CBT intervention will result in reductions in symptoms of PTSD, depression, and generalized anxiety for children. A secondary aim is to explore how the group-based TF-CBT intervention works. Identifying underlying mechanisms of the treatment may promote the effectiveness of the treatment.

**2. Methods**

**2.1 Design**

A single-blind (outcome assessors), parallel-group randomized clinical trial (RCT) is designed to evaluate the effectiveness of the group-based TF-CBT intervention. Trial recruitment started on the 2nd of November 2019. Completion of follow-up and closure of the data set is planned for December 2021. The study protocol has received ethics approval from Chinese Ethics Committee of Registering Clinical Trials (ChiECRCT-20180191) and was registered under the Chinese Clinical Trial Registry (ChiCTR1900027131).

**2.2 Participants’ eligibility and procedure**

Recruitment will be conducted at primary schools in Henan Province, China. To recruit adequate participants, a 1-hour overview of our program’s approach and its rationale will be presented to all stakeholders (children, guardians, the local education government, and school personnel). Following their agreement, a self-report questionnaire, including a range of potentially traumatic events and symptoms of PTSD (the PTSD Checklist-5), will be issued if children agree to participate in the study. We will use multiple methods to promote recruitment and retention, such as communicating regularly with stakeholders, providing gifts to participants at study completion, and ongoing reminder calls.

Children are eligible to participate if they 1) aged between 9 and 12 years old; 2) exposed to at least one traumatic event (> one month); 3) meet full PTSD or partial PTSD diagnostic criteria of the PTSD Checklist-5 (the PCL-5); 4) written informed consent will be obtained from himself and their parents. Exclusion criteria included: 1) severe psychopathology requiring urgent medical attention; 2) moderate to high suicide risk (e.g., intent or plan to attempt suicide in the near future); 3) showed receptive or expressive language difficulties (written or spoken); 4) current receipt of other mental health treatment, other than treatment as usual (TAU);

Based on results from our pilot randomized controlled trial, we estimated that the sample size would provide over 80% power to detect an effect size of 0·38 at a two-sided 5% significance level for the primary outcome at posttreatment. 110 participants per group were needed. Given an anticipated dropout rate of 30%, our target randomized controlled trial sample size was 286 in this study (calculated using G*Power 3·1).

**2.3 Randomization and masking**

Eligible children will be randomly allocated (1:1) to the TF-CBT group or the WL group. A random list is generated by using a computer-generated random sequence of numbers in the randomly sized block (2, 4, 6), stratified by PTSD severity (full PTSD or partial PTSD), and recruitment school sites. An investigator, who is not involved in subject recruitment, is responsible for generating this list and placing it in sequentially numbered, opaque, sealed envelopes. Two independent support staffs open a sealed envelope and record the group list.

**2.4 Intervention**

About one year before this RCT study, a pilot study was completed and the group-based TF-CBT was applied on 87 children in three primary schools. Based on this experience, various adaptations were made to enhance the contextual acceptability and feasibility of this intervention, such as the use of familiar games and culturally appropriate stories. Additionally, the delivery of individual sessions was modified from a late phase (after the completion of group session 6) to a middle phase (after the completion of group session 3).

Participants, in the TF-CBT group, will receive a 10-12 session, manualized, and culturally modified, the group TF-CBT intervention. This intervention comprises 7 weekly group sessions (each lasting up about 50 minutes) and 3-5 weekly individual sessions (each ranging from 30-50 minutes). The group-based TF-CBT intervention is divided into three phases: 1) stabilization and skills building (sessions 1-3: psychoeducation, relaxation, and emotion regulation); 2) exposure and cognitive processing of the trauma (sessions 4-6: cognitive coping, cognitive processing, and seeking social support); 3) fostering safety and future development (session 7).

The waiting list (WL) group will receive the TF-CBT treatment after they complete the 6-month follow-up assessment. During the waiting period, they do not receive any forms of intervention. However, arrangements are made for participants who become distressed during the waiting period. They will be withdrawn from the program for immediate intervention.

**2.4 Training and Supervision**

All therapy sessions are delivered by 15 undergraduate students recruited from Henan Normal University. Applicants can sign up if they: 1) ranked in the top 50 percent in cumulative GPA; 2) can regularly provide TF-CBT services one hour per week. 3) are willing to learn TF-CBT. Once they start to participate in the study, they will receive 3-day TF-CBT training and weekly supervision from manual developers from the Center for Behavioral Health, Beijing Normal University.

**2.4.1 Training**

Three full-day training courses will be conducted by the manual developers from the Center for Behavioral Health, Beijing Normal University. To further confirm that each trainee has a good understanding of the principles and methods of TF-CBT, didactic and active learning activities are included in the training workshop, such as small group discussions, role-plays, behavior rehearsals, and practice of techniques.

The training courses are divided into three stages. The first stage mainly introduces the information about trauma and PTSD, the development (theoretical rationale, key components, and effectiveness findings) of group-based TF-CBT intervention. The second stage focuses on teaching trainees how to deliver a 7-session group intervention. This includes 60 minutes of practice demonstration and 30 minutes of simulated training. After each simulation training, the trainer and trainees will discuss the advantages and disadvantages of simulation training. The third stage focuses on teaching trainees how to deliver case counseling. This includes a series of counseling skills, such as trauma narrative, identifying and combating negative thoughts, paraphrasing, selective attention, and punishment.

**2.4.2 Supervision**

To monitor treatment fidelity, supervisors will meet with trainees online each week after the initial training workshop that is discussed above is completed. Before the intervention, the consultation content mainly includes: 1) dealing with logistical issues (e.g., where and when to hold groups); 2) discussion of how to use training manuals and supporting materials; 3) discussion of session goals and content fidelity (trainees were expected to reach 80% fidelity in each session); 4) conducting didactic preparation for upcoming sessions; 5) providing psychological support. During the intervention period, trainees are requested to provide the former two-session video recordings, and the performance feedback, including content fidelity, implementation problems as well as improvement suggestions, were also recorded by supervisors when watching videos. After that, supervisors and trainees jointly discuss by phone or video. Teleconferences typically include:1) Reviewing content fidelity. For unachieved goals, supervisors and trainees will discuss whether supplements are needed in the following session; 2) discussion of implementation barriers and the solutions; 3) discussion of the potential difficulty and suggestions for improvement. 4) previewing the activity flow and session goals for the next session.

**2.5 Assessment**

All participants will receive assessments before randomization (t_1_), 4 weeks during the intervention phase (t_2_), 2 weeks posttreatment (t_3_), 3-month (t_4_) and 6-month post-end-of-treatment follow-up assessments (t_5_) (see fig 1). The time of two weeks after the treatment was the primary endpoint. Assessments were performed by trained assessors, who were masked to condition assignment of children. For an elaborate overview of study outcome measures, see Table 1.

Table 1 Overview of study outcome measures.

| Type of variable | Domain/Concept | Instrument | Items | Source | | | Assessment | | | | |
| --- | --- | --- | --- | --- | --- | --- | --- | --- | --- | --- | --- |
|  |  |  |  | I | C | T | t_1_ | t_2_ | t_3_ | t_4_ | t_5_ |
| Sociodemographic characteristics | Demographics |  |  | × | × |  | × |  |  |  |  |
|  | Traumatic events | PTSD- RI-5 part1 | 13 | × | × |  | × |  |  |  |  |
| Primary and secondary outcomes | PTSD | PTSD- RI-5 part2 | 27 | × | × |  | × | × | × | × | × |
|  |  | PCL-5 | 20 | × | × |  | × | × | × | × | × |
|  | Depression | CDI-S | 10 | × | × |  | × | × | × | × | × |
|  | Generalized Anxiety | The subscale of SCARED | 9 | × | × |  | × | × | × | × | × |
| Potential Mediators and Moderators | Cognitive | CERS-S | 18 | × | × |  | × | × | × | × | × |
|  |  | TMQQ | 11 | × | × |  | × | × | × | × | × |
|  | Emotion | CERS-S | 18 | × | × |  | × | × | × | × | × |
|  | Behavior | BADS-SF | 9 | × | × |  | × | × | × | × | × |
|  |  | SSBS | 22 | × | × |  | × | × | × | × | × |
|  | Social support | SSS-C | 24 | × |  |  |  | × | × |  |  |
| Treatment characteristics | Therapeutic alliance | TASC-r/TAST | 12 | × |  | × |  |  | × |  |  |
|  | Intervention satisfaction | CSQ-8 | 8 | × |  |  |  |  | × |  |  |

Abbreviations: Intervention group (I), Control group(C), therapist(T), t_1_ Baseline assessment, t_2_ 4 weeks during the intervention phase, t_3_ Posttreatment assessment, t_4_ 3-month follow-up assessment, t_5_ 6-month follow-up assessment.

**2.5.1 Primary outcome measures**

***UCLA PTSD Reaction Index for DSM-5 (PTSD-RI-5)*** is a self-report instrument for screening trauma exposure and assessing DSM-5 PTSD symptoms.^16^ Part one contains 13 potentially traumatic experiences. For each type of traumatic event, respondents are asked to state whether they are a victim, witness, or learned/heard about the trauma. Part two consists of 27 items to assess PTSD symptoms and 4 additional items to assess the dissociative subtype based on the DSM-5. Children rate the frequency of symptoms based on a 5-point Likert scale (from 0 = none of the time to 4 = most of the time). Items assessing PTSD symptoms are classified into 5 questions for Criterion B (intrusion), 2 questions for C (avoidance), 7 questions for D (negative alterations in cognitions and mood), and 6 questions for E (alterations in arousal and reactivity). Total PTSD scores are summed by B, C, D, and E subscale scores. Higher scores indicate more PTSD severity. In this study, PTSD-RI-5 is the main diagnostic tool to assess PTSD severity.

**2.5.2 Secondary outcome measure**

***PTSD Checklist-5（PCL-5）***is a 20-item self-report scale to assess the presence and severity of PTSD symptoms.^17^ Items are rated on a 5-point Likert scale from “not at all” (0) to “extremely” (4). There are two ways to be diagnosed with full PTSD: 1) children have a total score that is greater than or equal to 33. 2) children endorse at least moderate severity for each of the four symptom clusters, which means having one or more B items (items 1–5), one or more C items (items 6–7), two or more D items (items 8–14) and two or more E items (items 15–20) (Weathers et al., 2013). If a participant could not meet the DSM-5 PTSD criterion but endorsed three of four symptom clusters, he/she would be diagnosed with partial PTSD. The Chinese version of PCL-5 was adapted by translation and back translation, and has demonstrated good psychometric properties in Chinese children who suffer from trauma.^18^ In this study, PCL-5 is the main screening tool aiming to recruit children who meet the inclusion criteria of PTSD/ partial PTSD. In addition, it will be used to assess changes in PTSD symptoms over time.

***Children Depression Inventory-Short (CDI-S)*** is used to assess the severity of depressive symptoms for children. The CDI-S has 10 items, and each item is presented as a series of three phrases. Respondents are asked to select the phrase that best represents how they feel (e.g., “I am sad once in a while” / “I am sad many times” / “I am sad all the time”). Higher scores indicate more depression severity. High internal consistency (Cronbach’s α = 0·84) has been shown.^19^

***The Screen for Child Anxiety Related Emotional Disorders (SCARED)*** is a self-report scale to assess anxiety symptoms in children and youth. This includes 41 items that are divided into five specific anxiety disorders: panic disorder, generalized anxiety disorder, separation anxiety disorder, social phobic disorder, and school avoidance. Children rate each symptom’s frequency using a 3-point scale: 0 (not true or hardly ever true) to 2 (very true or often true). In this study, the subscale of SCARED (9 items) is selected to assess children’s generalized anxiety disorder. ^20^

**2.5.3 Potential Mediators and Moderators**

***Cognitive Emotional Regulation Scale-Short (CERS-S)*** is designed to assess an individual’s cognitive emotion regulation strategies for stressful events. The 18-item scale is divided into 9 dimensions: self-blame, other-blame, rumination, catastrophizing, positive refocusing, planning, positive reappraisal, acceptance, and putting into perspective. Each item is rated on a 5-point Likert scale ranging from 1 “Never” to 5 “Always”. A higher subscale score indicates greater use of a specific cognitive strategy. The CERS-S has shown good reliability and validity.^21^

***The Trauma Memory Quality Questionnaire (TMQQ)*** is an 11-item scale assessing the quality of the memories for traumatic events. The self-report scale inquires children the way they remember traumatic events. For example, “My memories of the frightening event are mostly pictures of images.” Children evaluate each item on a 4-point Likert scale (“1=Disagree a lot” to 4= “Agree a lot”). Good internal consistency (Cronbach’s α > 0·76) has been shown in the TMQQ.^22^

***Behavioral Activation for Depression Scale-Short Form (BADS-SF)*** is used to assess the changes in activation and avoidance behavior. 9 items are rated on a 7-point Likert scale ranging from 0 “not at all” to 6 (completely). Higher total scores indicate higher activity levels and lower avoidant levels. Good reliability (Cronbach’s α = 0·71) has been shown in BADS-SF.^23^

***Seek Safe Behavior Scale (SSBS)*** is a self-reported scale to assess an individual’s behavior strategies to prevent a feared outcome after traumatic events.^24^ 22 items are divided into two subscales: high alter strategy and effective monitoring. Excellent internal consistency (Cronbach’s α = 0·90) has been shown.^25^

***Social Support Scale for Children (SSS-C)*** is a 24-item scale assessing children’s perceptions of social support from parents, classmates, teachers, and close friends.^26^ Children are asked to decide which of two statements is more like him/her and whether the statement is “really true for me” or “sort of true for me”. Each item is rated on a 4-point Likert scale. A higher score indicates a higher level of perceived support. Good reliability (Cronbach's α = 0·83) has been shown in the previous study.^27^

**2.5.4 Treatment characteristics**

***The Client*** ***Satisfaction Questionnaire‐8 (CSQ-8)*** is used to assess the level of satisfaction with intervention.^28^ This scale consists of 8 items with a 4-point [Likert scale](https://www.sciencedirect.com/topics/medicine-and-dentistry/likert-scale). The scores, 8-20, 21-26, 27-32, indicate low level, medium level, and high level of satisfaction, respectively. The high internal consistency of this scale (Cronbach's α = 0·92) has been found.^29^

***Therapeutic Alliance Scale for Children, Revised (TASC-r)*** is one of the most commonly used alliance measures for children and adolescents.^30^ The TASC-r includes 12 items that comprise the affective bond (e.g., “I like my therapist”) and client-therapist collaboration on therapeutic tasks and goals (e.g., “I work with my therapist on solving my problems”). All items are rated on a 4-point Likert scale (not at all to very much). High internal consistency (Cronbach's α = 0·91) has been shown in previous studies.^31^

***Therapeutic Alliance Scales for Therapists (TASCT)*** is used to assess the therapist’s alliance.^31^ It is a parallel version of the TASC-r developed for therapists to use. Therapists rate their evaluation of the clients’ experience of the alliance on a 4-point Likert scale (e.g., “The child likes spending time with you, the therapist”). High reliability (Cronbach's α = 0·91) has been shown in the previous study.^32^

***Content fidelity*** is assessed by the Content Fidelity Checklist. This checklist was developed on the treatment components of TF-CBT by the treatment developers. In this checklist, items are rated as either “present” or “absent” to indicate whether some content is covered during a session. All “present” responses are added up and divided by the total number of items to obtain the average fidelity for each session. An acceptable level of fidelity for each session is a score of 80% or above.^33^ All therapy sessions will be video-recorded, and each session is coded by trained TF-CBT master students.

**2.6 Evaluation of adverse events**

To assess the participants’ adverse reactions, regular interviews were conducted with children, parents, and teachers. If the adverse reactions had a significant negative impact on participants’ psychological, physical, and social distress, the intervention would be stopped immediately.

**2.7 Data analysis**

Statistical analyses will be performed by an independent statistician who has not been involved in assessing the eligibility of participants, assigning individuals to treatment conditions, administering treatment, collecting follow-up assessment data, or inputting data. An intent-to-treat (ITT) approach will be adopted in this study. Missing data will be imputed 5 times for each missing item by multiple imputation procedures using R package mice 3·6·0. Missing data of intervention characteristics (including the Client Satisfaction, therapeutic Alliance) are not imputed.

To further analyze the longitudinal changes in outcomes between the TF-CBT group and the WL group over time, linear mixed models will be employed. Cohen's d is calculated to assess the magnitude of intervention effects. To explore effect of moderation, children’s demographics, traumatic events, social support, therapeutic alliance, and intervention satisfaction are used. Additionally, mediation analyses will be conducted to investigate whether cognitive-emotional regulation, trauma memory quality, behavioral activation, and seeking safe behavior strategies are mediators in the association between the group TF-CBT intervention and children’s PTSD symptoms.

**2.8 Data management**

The data monitoring committee, comprised of professors and doctors from the school of social development and public policy, Beijing Normal University, will be responsible for the oversight of data management of the trial. The role of the data monitoring committee is to review the statistical analysis plan, research questionnaire, and monitor data collection. Researchers will keep information obtained in this study confidential except as required by law. All participant questionnaires, records, informed consent forms, and all other related materials will be properly stored by the principal investigator. After the study, all the data will be double independently input by a professional data entry company to ensure data accuracy.

**Patient and public involvement**

The patients and the public will not be involved in the design, assessment, or conduction of this trial. Feedback will be sought from all participants at the end of the study to assess the burden of intervention and to help develop future trials.

**Ethics and dissemination**

Chinese-language informed consent will be obtained from all participants before the study begins. Any important modifications to this study protocol will be reported to the ethics committee of both institutions and the trial registries. All procedures will be conducted according to the Declaration of Helsinki. There are no data currently available for this paper as it is a protocol. However, the data will be made available in an open repository within six months after the trial is complete (http://cbh.bnu.edu.cn/). The findings of this study will be actively disseminated through peer-reviewed journals, conference presentations, social media, the internet, and community engagement activities.

**Reference**

1. Bolton D, Oryan D, Udwin O, Boyle S, Yule W. The long‐term psychological effects of a disaster experienced in adolescence: II: general psychopathology. *J Child Psychol Psychiatry* 2000; **41**: 513-23.

2. Zhai Y, Liu K, Zhang L, et al. The relationship between post-traumatic symptoms, parenting style, and resilience among adolescents in Liaoning, China: a cross-sectional study. *PloS One* 2015; **10**: e0141102.

3. Khamis V. Post-traumatic stress disorder among school age Palestinian children. *Child Abuse Negl* 2005; **29**: 81-95.

4. Patel V, Xiao S, Chen H, et al. The magnitude of and health system responses to the mental health treatment gap in adults in India and China. *Lancet* 2016; **388**: 3074-84.

5. Chisholm D, Sweeny K, Sheehan P, et al. Scaling-up treatment of depression and anxiety: a global return on investment analysis. *The Lancet Psychiatry* 2016; **3**: 415-24.

6. Resick PA, Wachen JS, Dondanville KA, et al. Effect of group vs individual cognitive processing therapy in active-duty military seeking treatment for posttraumatic stress disorder: A randomized clinical trial. *JAMA Psychiatry* 2017; **74**: 28-36.

7. Foa EB, Hembree EA, Cahill SP, et al. Randomized trial of prolonged exposure for posttraumatic stress disorder with and without cognitive restructuring: outcome at academic and community clinics. *J Consult Clin Psychol* 2005; **73**: 953-64.

8. Lely JCG, Smid GE, Jongedijk RA, Knipscheer JW, Kleber RJ. The effectiveness of narrative exposure therapy : A review, meta-analysis and meta-regression analysis. *Eur J Psychotraumatol* 2019; **10**: 1550344.

9. Cohen JA, Mannarino AP. Trauma‐focused cognitive behavioural therapy for children and parents. *Child and Adolescent Mental Health* 2008; **13**: 158-62.

10. Mavranezouli I, Megninviggars O, Daly C, et al. Psychological and psychosocial treatments for children and young people with post-traumatic stress disorder: a network meta-analysis. *J Child Psychol Psychiatry* 2019.

11. Deblinger E, Pollio E, Dorsey S. Applying trauma-focused cognitive–behavioral therapy in group format. *Child Maltreat* 2016; **21**: 59-73.

12. Yasui M, Pottick KJ, Chen Y. Conceptualizing culturally infused engagement and its measurement for ethnic minority and immigrant children and families. *Clinical Child and Family Psychology Review* 2017; **20**: 250-332.

13. Stein BD, Jaycox LH, Kataoka SH, et al. A mental health intervention for schoolchildren exposed to violence: a randomized controlled trial. *JAMA* 2003; **290**: 603-11.

14. Calderón L. The cognitive impact of sexual abuse and PTSD in children: a neuropsychological study. *J Child Sex Abus* 2013; **22**: 625-38.

15. Lindebø Knutsen M, Sachser C, Holt T, Goldbeck L, Jensen TK. Trajectories and possible predictors of treatment outcome for youth receiving trauma-focused cognitive behavioral therapy. *Psychological trauma : theory, research, practice and policy* 2019; **12**: 336–46.

16. Kaplow JB, Rolon-Arroyo B, Layne CM, et al. Validation of the UCLA PTSD Reaction Index for DSM-5: a developmentally informed assessment tool for youth. *J Am Acad Child Adolesc Psychiatry* 2020; **59**: 186-94.

17. Weathers FW, Litz BT, Keane TM, Palmieri PA, Marx BP, Schnurr PP. The PTSD checklist for DSM-5 (PCL-5). *Department of Veterans Affairs, National Center for Posttraumatic Stress Disorder* 2013.

18. Li J, Zhang W, Chen W, et al. Applications of the Chinese version of the primary care PTSD screen for DSM-5 (PC-PTSD-5) for children. *J Affect Disord* 2019; **254**: 109-14.

19. de la Vega R, Racine M, Sanchez-Rodriguez E, et al. Psychometric properties of the short form of the Children's Depression Inventory (CDI-S) in young people with physical disabilities. *J Psychosom Res* 2016; **90**: 57-61.

20. Birmaher B, Brent DA, Chiappetta L, Bridge J, Monga S, Baugher M. Psychometric properties of the screen for child anxiety related emotional disorders (SCARED): a replication study. *J Am Acad Child Adolesc Psychiatry* 1999; **38**: 1230-6.

21. Garnefski N, Kraaij V. Cognitive emotion regulation questionnaire – development of a short 18-item version (CERQ-short). *Personality and Individual Differences* 2006; **41**: 1045-53.

22. Meiser-Stedman. R, Smith. P, Yule. W, Dalgleish T. The Trauma Memory Quality Questionnaire: Preliminary development and validation of a measure of trauma memory characteristics for children and adolescents. *MEMORY* 2007; **15**: 271-9.

23. Gonzalez-Roz A, Secades-Villa R, Muniz J. Validity evidence of the Behavioral Activation for Depression Scale-Short Form among depressed smokers. *Int J Clin Health Psychol* 2018; **18**: 162-9.

24. Ehring. T, Ehlers. A, Glucksman. E. Do Cognitive Models Help in Predicting the Severity of Posttraumatic Stress Disorder, Phobia, and Depression After Motor Vehicle Accidents? A Prospective Longitudinal Study. *Journal of Consulting and Clinical Psychology* 2008; **2008**: 219-30.

25. Alberici A, Meiser-Stedman R, Claxton J, et al. The Preliminary Development and Validation of a Trauma-Related Safety-Seeking Behavior Measure for Youth: The Child Safety Behavior Scale (CSBS). *J Trauma Stress* 2018; **31**: 643-53.

26. Gordon-Hollingsworth AT, Thompson JE, Geary MA, Schexnaildre MA, Lai BS, Kelley ML. Social Support Questionnaire for Children. *Measurement and Evaluation in Counseling and Development* 2017; **49**: 122-44.

27. Jackson Y, Warren JS. Appraisal, social support, and life events: predicting outcome behavior in school-age children. *Child Development* 2000; **71**: 1441-57.

28. Attkisson CC, Zwick R. The client satisfaction questionnaire. Psychometric properties and correlations with service utilization and psychotherapy outcome. *Evaluation & Program Planning* 1982; **5**: 233-7.

29. Kelly PJ, Kyngdon F, Ingram I, Deane FP, Baker AL, Osborne BA. The client satisfaction questionnaire-8: psychometric properties in a cross-sectional survey of people attending residential substance abuse treatment. *Drug Alcohol Rev* 2017.

30. Ormhaug SM, Jensen TK, Wentzel-Larsen T, Shirk SR. The therapeutic alliance in treatment of traumatized youths: relation to outcome in a randomized clinical trial. *J Consult Clin Psychol* 2014; **82**: 52-64.

31. Accurso EC, Garland AF. Child, caregiver, and therapist perspectives on therapeutic alliance in usual care child psychotherapy. *Psychol Assess* 2015; **27**: 347-52.

32. Ormhaug SM, Shirk SR, Wentzel-Larsen T. Therapist and client perspectives on the alliance in the treatment of traumatized adolescents. *Eur J Psychotraumatol* 2015; **6**: 27705.

33. Breitenstein SM, Gross D, Garvey CA, Hill C, Fogg L, Resnick B. Implementation fidelity in community-based interventions. *Res Nurs Health* 2010; **33**: 164-73.

# Supplementary Results

## Table S1. Baseline characteristics by completion of three timepoint assessments.

|  | Completion (n=234) | Incompletion (n=13) | Stat^*^ | *P* |
| --- | --- | --- | --- | --- |
| **Age, mean (SD)** | 10·41 (0·88) | 10·18 (1·08) | -0·90 | 0·371 |
| **Gender** |  |  |  |  |
| boy | 137 (58·55%) | 7 (63·64%) | 0·00 | 0·983 |
| girl | 97 (41·45%) | 4 (36·36%) |  |  |
| **School grade** |  |  |  |  |
| 3 | 19 (8·12%) | 2 (18·18%) | 1·67 | 0·434 |
| 4 | 93 (39·74%) | 3 (27·27%) |  |  |
| 5 | 122 (52·14%) | 6 (54·55%) |  |  |
| **Parent’s marital status** |  |  |  |  |
| Married or remarried | 205 (87·61%) | 9 (81·82%) | 0·01 | 0.92 |
| Single or widowed | 29 (12·39%) | 2 (18·18%) |  |  |
| **Boarders** |  |  |  |  |
| yes | 91 (39·74%) | 2 (18·18%) | 1·25 | 0·264 |
| No | 138 (60·26%) | 9 (81·82%) |  |  |
| **PTSD-RI-5 PTSD, M(SD)** | 40·45 (17·19) | 35.45 (17·98) | -0·99 | 0·325 |
| **Depression (CDI-S), M(SD)** | 7·85 (4·32) | 5.55 (3·50) | -1·82 | 0·070 |
| **Generalized anxiety disorder (the subscale of SCARED), M(SD)** | 9·41 (4·21) | 7.09 (3·81) | -1·88 | 0·062 |
| **PCL-5 PTSD, M(SD)** | 42·54 (13·22) | 42.95 (14·24) | 0·10 | 0·918 |

PTSD-RI-5=PTSD Reaction Index for DSM-5 (total score range 0-80; higher scores indicated severe posttraumatic stress disorder severity); CDI-S=Children Depression Inventory-Short (total score range 0-20; higher scores indicated severe depression severity); SCARED=The Screen for Child Anxiety Related Emotional Disorders (subscale score range 0–18; higher scores indicated severe generalized anxiety disorder severity); PCL-5=PTSD Checklist-5 (total score range 0-80; higher scores indicated severe posttraumatic stress disorder severity). ^*^ t value of *t* test or χ^2^ value of χ^2^ test.

## Table S2. The content fidelity for each group session.

|  | N | Mean | SD |
| --- | --- | --- | --- |
| Psychoeducation | 15 | 0·90 | 0·11 |
| Relaxation | 15 | 0·87 | 0·08 |
| Emotional management and regulation | 15 | 0·82 | 0·11 |
| Cognitive coping | 15 | 0·86 | 0·14 |
| Cognitive processing | 15 | 0·88 | 0·06 |
| Social support | 15 | 0·97 | 0·08 |
| Safety plan | 15 | 0·97 | 0·05 |

## Table S3. Primary outcomes at three assessment points for different PTSD severity groups.

| Severity of PTSD-RI-5 | PCPI (n = 118) | | | TAU (n = 116) | | | Cohen’s d | 95% CI | *P* value |
| --- | --- | --- | --- | --- | --- | --- | --- | --- | --- |
|  | Mean | SD | 95% CI | Mean | SD | 95% CI |  |  |  |
| Low scores (< 35 points) | n = 42 | | | n = 41 | | |  |  |  |
| Baseline | 21·04 | 7·62 | 18·74 - 23·35 | 22·51 | 7·50 | 20·22 - 24·81 |  |  |  |
| Posttreatment | 23·07 | 15·11 | 18·50 - 27·64 | 24·32 | 18·32 | 18·71 - 29·93 | 0·01 | -0·42 - 0·44 | 0·974 |
| 3-month follow up | 24·93 | 22·63 | 18·09 - 31·78 | 25·06 | 19·63 | 19·06 - 31·07 | 0·04 | -0·39 - 0·47 | 0·867 |
| High scores (≥ 35 points) | n = 76 | | | n = 75 | | |  |  |  |
| Baseline | 49·90 | 10·04 | 47·64 - 52·16 | 51·54 | 12·54 | 48·71 - 54·38 |  |  |  |
| Posttreatment | 35·35 | 16·84 | 31·57 - 39·14 | 47·37 | 17·89 | 43·32 - 51·42 | -0·66 | -0·99 - -0·33 | < 0·001 |
| 3-month follow up | 33·46 | 17·53 | 29·51 - 37·40 | 37·36 | 17·94 | 33·30 – 41·42 | -0·20 | -0·52 - 0·12 | 0·221 |

Abbreviations: PTSD, Posttraumatic Stress Disorder; PCPI, the Power up Children’s Psychological Immunity; TAU, Treatment as Usual; PTSD-RI-5=UCLA PTSD Reaction Index for DSM-5.

## Table S4. Sensitivity analyses for all participants, participants without missing values, and participants with full PTSD.

|  | N | Baseline level  Mean (SD) | Adjusted difference between PCPI and TAU | 95% CI | *P* Value |
| --- | --- | --- | --- | --- | --- |
| **PTSD (PTSD-RI-5)** |  |  |  |  |  |
| All participants (after multiple imputation) | 234 | 40·45 (17·19) | -7·52 | -12·12 - -2·93 | 0·001 |
| Participants without missing values in PTSD-RI-5 | 204 | 40·50 (17·56) | -6·40 | -10·82 - -1·98 | 0·005 |
| Participants with full PTSD | 193 | 42·66 (17·05) | -5·80 | -10·89 - -0·72 | 0·026 |
| **Depression (CDI-S)** |  |  |  |  |  |
| All participants (after multiple imputation) | 234 | 7·85 (4·32) | -1·76 | -2·83 - -0·69 | 0·001 |
| Participants without missing values in CDI-S | 220 | 7·99 (4·34) | -1·58 | -2·50 - -0·65 | 0·001 |
| Participants with full PTSD | 193 | 8·34 (4·36) | -1·62 | -2·77 - -0·46 | 0·006 |
| **Generalized anxiety disorder (the subscale of SCARED)** |  |  |  |  |  |
| All participants (after multiple imputation) | 234 | 9·41 (4·21) | -1·24 | -2·32 - -0·16 | 0·024 |
| Participants without missing values in SCARED | 222 | 9·55 (4·20) | -1·20 | -2·23 - -0·17 | 0·023 |
| Participants with full PTSD | 193 | 9·78 (4·22) | -1·02 | -2·22 - 0·18 | 0·098 |
| **PTSD (PCL-5)** |  |  |  |  |  |
| All participants (after multiple imputation) | 234 | 42·54 (13·22) | -8·58 | -13·59 - -3·58 | 0·001 |
| Participants without missing values in PCL-5 | 206 | 42·43 (13·16) | -8·98 | -13·92 - -4·04 | <0·001 |
| Participants with full PTSD | 193 | 45·84 (11·81) | -6·84 | -12·42 - -1·25 | 0·017 |

Abbreviations: PCPI, the Power up Children’s Psychological Immunity; TAU, Treatment as Usual; PTSD-RI-5= PTSD Reaction Index for DSM-5 (total score range 0-80; higher scores indicated severe posttraumatic stress disorder severity); CDI-S=Children Depression Inventory-Short (total score range 0-20; higher scores indicated severe depression severity); SCARED=The Screen for Child Anxiety Related Emotional Disorders (subscale score range 0–18; higher scores indicated severe generalized anxiety disorder severity); PCL-5=PTSD Checklist-5 (total score range 0-80; higher scores indicated severe posttraumatic stress disorder severity).
